# Supplementary material for: Uncovering the mechanisms of synergistic drug combinations in non-small cell lung cancer through metagene-based classification
Source: PLoS One. 2026 May 12;21(5):e0343902. doi: 10.1371/journal.pone.0343902 (PMC13166920; doi:10.1371/journal.pone.0343902)
Supplement: S1 Text — (DOCX) [file pone.0343902.s001.docx]

**Supporting Information S1 Text**

**Uncovering the mechanisms of synergistic drug combinations in non-small cell lung cancer through metagene-based classification**

Comkrit Lomloy, Piyanut Ratphibun Yamashita, Teerasit Termsaithong, Teeraphan Laomettachit

**S1 Table.** All clusters with an average synergy score greater than the third quartile (Q3) of their respective synergy score type. The highlighted rows represent the specific heights chosen for further analysis in the main text.

| Height at which the dendogram was cut (*h*) | Cluster ID | Number of drug pairs in the cluster | HSA | ZIP | Bliss | Loewe | Drug with the highest frequency | Number of drug pairs with the most frequent drug |
| --- | --- | --- | --- | --- | --- | --- | --- | --- |
| 148.48 | 26 | 17 | 4.31 ± 5.59 |  |  |  | Crizotinib | 13 |
| 261.69 | 4 | 24 | 4.28 ± 5.6 |  | 3.81 ± 6.22 |  | Dasatinib | 24 |
| 115.69 | 28 | 11 | 7.88 ± 5.11 | 3.38 ± 6.75 | 8.24 ± 5.9 |  | Dasatinib | 11 |
| 156.53 | 8 | 24 | 4.06 ± 4.44 |  |  |  | Erlotinib | 8 |
| 104.12 | 27 | 12 | 5.14 ± 4.1 |  | 4.64 ± 4.42 |  | Erlotinib | 4 |
| 102.69 | 34 | 10 |  |  |  | 0.01 ± 2.9 | Gefitinib | 5 |
| 88.06 | 14 | 11 |  |  | 4.31 ± 9.72 |  | Mitotane | 11 |
| 66.86 | 16 | 10 |  | 2.58 ± 6.58 | 5.38 ± 9.54 |  | Mitotane | 10 |
| 133.28 | 19 | 11 | 8.37 ± 6.48 | 7.02 ± 7.39 | 7.74 ± 6.48 |  | Paclitaxel | 10 |
| 155.46 | 22 | 18 | 5.11 ± 5.92 | 2.6 ± 6.81 | 5.78 ± 5.71 |  | Pazopanib | 18 |
| 83.97 | 55 | 15 | 4.8 ± 6.37 | 3.21 ± 7.22 | 5.4 ± 6.13 |  | Pazopanib | 15 |
| 348.84 | 2 | 56 | 3.99 ± 7.38 |  | 3.9 ± 7.51 |  | Quinacrine | 29 |
| 215.87 | 2 | 26 | 7.68 ± 8.02 | 5.14 ± 8.62 | 7.77 ± 8.12 |  | Quinacrine | 26 |
| 124.14 | 2 | 15 | 11.83 ± 7.65 | 9.71 ± 8.41 | 12 ± 7.78 | 4.83 ± 14.34 | Quinacrine | 15 |
| 78.19 | 69 | 10 | 3.93 ± 7.06 |  |  |  | Ruxolitinib | 10 |
| 190.5 | 20 | 14 |  | 3.92 ± 11.32 | 3.84 ± 12.59 |  | Vemurafenib | 11 |

**S1 Figure.** All drug combinations containing **dasatinib** (a) Metagene profiles (matrix *H*) of all drug combinations containing **dasatinib**. (b) Box plots comparing the synergy scores between the two groups. (This figure is identical to Fig. 6 in the main text and is included here for easier comparison with S2 Table.)

**S2 Table.** Comparison of two groups of drug combinations containing **dasatinib**.

| Group | HSA  (Mean ± SD) | ZIP  (Mean ± SD) | Bliss  (Mean ± SD) | Loewe  (Mean ± SD) |
| --- | --- | --- | --- | --- |
| 1 | 1.39 ± 3.93 | −3.42 ± 2.98 | 0.33 ± 3.45 | −6.58 ± 15.47 |
| 2 | 7.58 ± 5.76 | 3.28 ± 5.62 | 7.63 ± 5.64 | −9.59 ± 14.90 |
| p-value (t-test, one-sided) | 0.0003822* | 4.637E−5* | 2.603E−5* | 0.7123 |

| **a** | **b** |
| --- | --- |
| **** | **** |

**S2 Figure.** All drug combinations containing **paclitaxel** (a) Metagene profiles (matrix *H*) of all drug combinations containing **paclitaxel**. (b) Box plots comparing the synergy scores between the two groups.

**S3 Table.** Comparison of two groups of drug combinations containing **paclitaxel**.

| Group | HSA  (Mean ± SD) | ZIP  (Mean ± SD) | Bliss  (Mean ± SD) | Loewe  (Mean ± SD) |
| --- | --- | --- | --- | --- |
| 1 | 7.06 ± 6.88 | 6.65 ± 7.62 | 6.31 ± 7.19 | −9.18 ± 12.54 |
| 2 | −3.33 ± 4.17 | −3.20 ± 4.36 | −2.96 ± 4.31 | −16.43 ± 11.53 |
| p-value (t-test, one-sided) | 2.638E−6* | 2.054E−5* | 2.564E−5* | 0.04616* |

| **a** | **b** |
| --- | --- |
| **** | **** |

**S3 Figure.** All drug combinations containing **quinacrine** (a) Metagene profiles (matrix *H*) of all drug combinations containing **quinacrine**. (b) Box plots comparing the synergy scores between the two groups.

**S4 Table.** Comparison of two groups of drug combinations containing **quinacrine**.

| Group | HSA  (Mean ± SD) | ZIP  (Mean ± SD) | Bliss  (Mean ± SD) | Loewe  (Mean ± SD) |
| --- | --- | --- | --- | --- |
| 1 | 1.28 ± 4.13 | −1.55 ± 3.50 | 1.37 ± 4.16 | −13.24 ± 11.19 |
| 2 | 10.32 ± 8.12 | 9.03 ± 8.46 | 10.75 ± 8.01 | 4.17 ± 13.62 |
| p-value (t-test, one-sided) | 0.0001316* | 2.258E−5* | 7.627E−5* | 0.0001732* |

| **a** | **b** |
| --- | --- |
|  | **** |

**S4 Figure.** All drug combinations containing **crizotinib** (a) Metagene profiles (matrix *H*) of all drug combinations containing **crizotinib**. (b) Box plots comparing the synergy scores between the two groups.

**S5 Table.** Comparison of two groups of drug combinations containing **crizotinib**.

| Group | HSA  (Mean ± SD) | ZIP  (Mean ± SD) | Bliss  (Mean ± SD) | Loewe  (Mean ± SD) |
| --- | --- | --- | --- | --- |
| 1 | 2.77 ± 5.87 | −1.62 ± 5.42 | 2.98 ± 5.51 | −3.01 ± 7.67 |
| 2 | −2.17 ± 4.97 | −3.92 ± 4.48 | −2.37 ± 6.24 | −11.14 ± 12.85 |
| p-value (t-test, one-sided) | 0.00635* | 0.0929 | 0.007958* | 0.02367* |

| **a** | **b** |
| --- | --- |
|  | **** |

**S5 Figure.** All drug combinations containing **pazopanib** (a) Metagene profiles (matrix *H*) of all drug combinations containing **pazopanib**. (b) Box plots comparing the synergy scores between the two groups.

**S6 Table.** Comparison of two groups of drug combinations containing **pazopanib**.

| Group | HSA  (Mean ± SD) | ZIP  (Mean ± SD) | Bliss  (Mean ± SD) | Loewe  (Mean ± SD) |
| --- | --- | --- | --- | --- |
| 1 | 4.73 ± 5.74 | 2.34 ± 6.49 | 5.43 ± 5.51 | −4.73 ± 14.93 |
| 2 | −1.52 ± 5.65 | 0.06 ± 4.50 | −0.92 ± 5.64 | −17.75 ± 19.06 |
| p-value (t-test, one-sided) | 0.001885* | 0.061 | 0.001474* | 0.02154* |

| **a** | **b** |
| --- | --- |
|  | **** |

**S6 Figure.** All drug combinations containing **ruxolitinib** (a) Metagene profiles (matrix *H*) of all drug combinations containing **ruxolitinib**. (b) Box plots comparing the synergy scores between the two groups.

**S7 Table.** Comparison of two groups of drug combinations containing **ruxolitinib**.

| Group | HSA  (Mean ± SD) | ZIP  (Mean ± SD) | Bliss  (Mean ± SD) | Loewe  (Mean ± SD) |
| --- | --- | --- | --- | --- |
| 1 | 3.14 ± 6.87 | −0.24 ± 6.65 | 3.22 ± 6.32 | −1.78 ± 7.16 |
| 2 | 0.41 ± 4.44 | −1.53 ± 4.15 | 0.34 ± 4.93 | −10.46 ± 14.22 |
| p-value (t-test, one-sided) | 0.08483 | 0.2451 | 0.07283 | 0.02494* |

| **a** | **b** |
| --- | --- |
|  | **** |

**S7 Figure.** All drug combinations containing **erlotinib** (a) Metagene profiles (matrix *H*) of all drug combinations containing **erlotinib**. (b) Box plots comparing the synergy scores between the two groups.

**S8 Table.** Comparison of two groups of drug combinations containing **erlotinib**.

| Group | HSA  (Mean ± SD) | ZIP  (Mean ± SD) | Bliss  (Mean ± SD) | Loewe  (Mean ± SD) |
| --- | --- | --- | --- | --- |
| 1 | 5.32 ± 4.12 | 1.55 ± 3.06 | 4.19 ± 3.07 | 1.35 ± 7.17 |
| 2 | 4.53 ± 5.13 | 0.36 ± 5.06 | 4.16 ± 4.71 | −4.85 ± 10.39 |
| p-value (t-test, one-sided) | 0.3177 | 0.222 | 0.4917 | 0.033* |

| **a** | **b** |
| --- | --- |
|  | **** |

**S8 Figure.** All drug combinations containing **gefitinib** (a) Metagene profiles (matrix *H*) of all drug combinations containing **gefitinib**. (b) Box plots comparing the synergy scores between the two groups.

**S9 Table.** Comparison of two groups of drug combinations containing **gefitinib**.

| Group | HSA  (Mean ± SD) | ZIP  (Mean ± SD) | Bliss  (Mean ± SD) | Loewe  (Mean ± SD) |
| --- | --- | --- | --- | --- |
| 1 | 5.16 ± 3.43 | 0.80 ± 3.93 | 2.45 ± 4.56 | 2.63 ± 5.27 |
| 2 | 4.46 ± 4.36 | 0.97 ± 4.46 | 2.41 ± 4.48 | 0.03 ± 5.62 |
| p-value (t-test, one-sided) | 0.7035 | 0.4539 | 0.5116 | 0.9151 |

**S10 Table.** List of the top 200 contributing genes of Metagene 2.

| A2M  ABL2  ADGRB1  ADIPOQ  AFF3  AKT2  ALK  ALOX15  AREG  BMP2  BMP4  BMPR1B  BRAF  C3  CACNA1B  CCL21  CCL22  CCR7  CCR9  CD3G  CD40LG  CD44  CDH11  CHP2  CNR2  CRP  CXCL11  CXCL2  CXCL5  CXCR2  CYP4A11  DOCK1  DUSP1  DUSP2  DUSP4  DUSP6  DVL1  EDA  EGF  EGFR | EPO  ERBB2  ERBB3  ERBB4  FEV  FGF1  FGF10  FGF11  FGF12  FGF14  FGF16  FGF17  FGF18  FGF19  FGF2  FGF20  FGF21  FGF22  FGF3  FGF4  FGF5  FGF6  FGF7  FGF8  FGF9  FGFR1  FGFR2  FGFR4  FLT1  FLT4  FYN  FZD2  FZD4  FZD7  HBEGF  HGF  IFNA16  IFNA17  IFNA4  IFNB1 | IFNG  IGF1  IL12A  IL12B  IL18  IL18R1  IL23A  IL24  IL2RB  IL2RG  IL3  IL3RA  IL4  IL5  IL7  IL9  IL9R  INHBA  INHBE  IRF6  ITGA2B  ITGA5  ITK  JAK2  KITLG  LTA  LYN  MAPK10  MAPK11  MAPK8  MAPT  MMP1  NCAM1  NCKIPSD  NECTIN1  NGF  NOS3  NOTCH4  NOX1  NRG1 | NRG2  NRG4  NTRK2  NTRK3  OMD  ORM1  ORM2  OSM  PCSK5  PDGFA  PDGFC  PDGFD  PDGFRB  PDPK1  PDPN  PGR  PIK3CD  PIK3R2  PIK3R5  PLA2G3  PLA2G4A  PLA2G5  PLA2G6  PLCB1  PLCB2  PRKACG  PRKCG  PTGS1  PTGS2  PTK2  PTPN5  PTPN6  PTPN7  PTPRB  PTPRD  PTPRJ  PTPRM  PTPRR  PTPRU  PTX3 | RELN  RET  ROBO2  RPS6KA2  S1PR3  SDC2  SFRP2  SFRP4  SH2B3  SHC1  SHC2  SHC3  SHC4  SMO  SOCS3  SOS1  SPHK1  SPRY2  SRC  SRGAP3  STAB1  STAT4  STAT5B  STAT6  STK36  TGFA  TGFB3  THBS2  TNF  TNFRSF11B  TNFSF11  TNFSF15  TNR  TYK2  VEGFC  VWF  WNT11  WNT2  WNT2B  XCR1 |
| --- | --- | --- | --- | --- |
