# Supplementary material for: Uncovering the mechanisms of synergistic drug combinations in non-small cell lung cancer through metagene-based classification
Source: PLoS One. 2026 May 12;21(5):e0343902. doi: 10.1371/journal.pone.0343902 (PMC13166920; doi:10.1371/journal.pone.0343902)
Supplement: Attachment — Submitted filename: Response_to_Reviewers_PLOSONE2025_revision1.pdf [file pone.0343902.s006.pdf]

Response to Reviewers

Uncovering the mechanisms of synergistic drug combinations in non-small cell lung cancer through metagene-based classification

Black = Comments from the editor or reviewers
Red = Authors' responses
Black highlighted = Changes made to the manuscript

Editor's comment

Thank you for submitting your manuscript to PLOS ONE. After careful consideration, we feel that it has merit but does not fully meet PLOS ONE's publication criteria as it currently stands. Therefore, we invite you to submit a revised version of the manuscript that addresses the points raised during the review process.

We would like to thank the editor and the reviewers for taking the time to provide feedback on our work. We have carefully considered your suggestions and made the necessary changes to the manuscript. We believe that these revisions have significantly strengthened the clarity and quality of our work, and we hope that you will find the revised version much improved.

Reviewers' comments

Reviewer #1:

The study addresses the synergistic drug combinations that simultaneously target multiple signaling pathways to overcome anticancer drug resistance. The authors identified the metagene 2 as a driving force behind the effectiveness of the drug combinations by the interconnected RAS/MAPK and PI3K/AKT signaling pathways. This metagene-based framework provides a valuable resource for designing targeted multi-drug therapies for NSCLC. The subject is interesting and addresses the important issue of drug resistance in cancer therapy. The research design is sound, well conducted, and the manuscript is well-written and may be suitable for publication.

We appreciate the reviewer's positive feedback.

Reviewer #2:

In the current manuscript, the authors present methodologically, systems-level framework for understanding mechanistic drivers of drug synergy in the A549 Non-small cell lung cancer (NSCLC) cell line. By combining frameworks, Random Walk with Restart (RWR) and Graph-regularized Non-negative Matrix Factorization (GNMF), the authors derive pathway-level "metagene" signatures which enable interpretable drug combinations and mechanistic hypothesis generation. In previous studies, Cheng et al. demonstrated that analyzing drug–disease and drug–drug proximities within the human interactome can successfully predict synergistic combinations by exploiting the spatial geometry of disease modules (doi.org/10.1038/s41467-019-09186-x). Another study showed that propagating somatic mutation profiles over a protein–protein interaction network, followed by matrix factorization, reveals biologically meaningful tumor subtypes predictive of clinical outcomes (doi.org/10.1038/nmeth.2651;

doi.org/10.1093/bioinformatics/btaa1099). The present manuscript is conceptually consistent with these approaches but extends them through a more expressive metagene representation. This extension produces pathway-level signatures rather than topological proximity scores, enabling the mechanistic insight stated by the authors. The methods presented are well aligned with previous network-based stratification (NBS). The current study applies the same conceptual machinery to drug combination profiles, using an A549-specific cancer network and graph-regularized NMF to distill 2100-gene propagated vectors into 26 biologically coherent metagenes. Here, by identifying a dominant metagene (Metagene 2) whose constituent genes map to PI3K/AKT, RAS, and MAPK signaling, the authors provide a direct mechanistic rationale for why certain drug pairs (e.g., dasatinib–axitinib, lapatinib–paclitaxel, quinacrine–TKI combinations) exhibit strong synergy. This pathway-level interpretability fills an important gap in current drug-combination research and is within the scope of the journal. The manuscript can be improved based on the comments below:

We appreciate the reviewer's insights and have incorporated the suggested improvements to strengthen our manuscript, as shown below.

1. Although the authors note this in the Discussion, elaborating on how the framework might extend to other NSCLC subtypes or pan-cancer analyses would add depth to the conclusion.

We thank the reviewer for this suggestion. We agree that elaborating on the generalizability of our framework adds significant value to the study. In the revised manuscript, we have expanded the Discussion section to explicitly outline how the framework can be adapted for other NSCLC subtypes and pan-cancer analyses.

Lines 592-600:
Importantly, the framework is adaptable to other NSCLC subtypes and pan-cancer analyses, extending beyond the A549 lung adenocarcinoma cell line. The core workflow involves constructing a context-specific network by integrating gene mutation status from the cell line of interest. Therefore, extending this to other subtypes requires tailoring the generic network using mutation or transcriptomic profiles specific to those cell lines. Furthermore, the framework can facilitate pan-cancer analysis by aggregating propagated profiles from diverse cell lines into a single matrix for factorization. This approach will enable the identification of universal metagenes that represent fundamental synergy mechanisms conserved across cancer types, as well as metagenes unique to specific cancer subtypes.

2. Authors should provide clear justification for restricting analysis to drugs with inhibitory targets only and discuss potential implications for activators or stabilizers.

We appreciate this important question. We restricted our analysis to drugs with inhibitory mechanisms mainly because most drugs retrieved from the database are inhibitors, reflecting the dominant therapeutic strategy of suppressing oncogenic signaling pathways. In addition, incorporating activators or stabilizers would introduce conflicting signal directions, greatly increasing analytical complexity and complicating interpretation. We recognize that this exclusion is a limitation of the current approach, and future work should address this, for

example, by modeling bidirectional or context-dependent signaling to capture the interplay between pathway agonists and antagonists. We have added this discussion to our revised manuscript.

Lines 602-613:
A key limitation of the current framework is its restriction to drugs with inhibitory mechanisms. This choice was primarily motivated by the fact that most drugs retrieved from the database are inhibitors, reflecting the dominant therapeutic strategy of suppressing oncogenic signaling pathways. Focusing exclusively on inhibitory effects enables a clear, unidirectional interpretation of signal propagation within the cancer network, as drug action is consistently modeled as the suppression of protein activity. In contrast, including activators or stabilizers would introduce conflicting signal directions, which greatly increases analytical complexity. In addition, two drugs that share the same protein targets but one is an activator, and the other an inhibitor, would have the same propagation profile, which complicates interpretation. We recognize that this exclusion represents a limitation of the present approach. Future directions should address this by modeling bidirectional or context-dependent signaling to capture the interplay between pathway agonists and antagonists.

3. Please include FDR-adjusted p-values directly in Figs. 2 to 4, 6c and 7a.

We include the FDR-adjusted p-values in Figures 2, 3, and 6b as suggested.

For Fig. 4, we chose to report the statistical significance in the figure caption and main text instead of on the plot itself to keep visual clarity. We conducted statistical analysis to confirm that the signal for Metagene 2 is significantly higher than that of all other metagenes (one-sided Wilcoxon signed-rank test, FDR-adjusted p-value < 0.05). This information has been included in both the main text and the caption of Figure 4.

For Fig. 7a, to maintain visual clarity and prevent overcrowding the plot, we use an asterisk (*) to indicate statistical significance. The asterisk marks the single condition (vemurafenib under the Bliss model) that showed synergy scores significantly above zero. We have revised the figure caption to explicitly clarify this.

Caption of Fig. 7a:
The asterisk (*) denotes that the Bliss scores of drug combinations involving vemurafenib are the only condition with synergy scores significantly higher than zero (one-sided one-sample Wilcoxon signed-rank test, FDR-adjusted p-value < 0.05).

Reviewer #3:

We appreciate the reviewer's feedback and have made the recommended revisions to improve our manuscript, as shown below.

1- The introduction part is lengthy and and be trimmed.
We have significantly shortened the introduction in the revised manuscript.

2- The discussion part should be strengthen and the authors should highlight the new aspects of the current study, compare this with the similar manuscript regarding the issue.

We thank the reviewer for this suggestion. In the revised Discussion, we explicitly compare our method with topological proximity models (e.g., Cheng et al., 2019), emphasizing how our use of network propagation (RWR) captures global diffusion and indirect effects that local distance metrics may overlook. We also highlight the novelty of adapting matrix factorization to drug combination profiles.

Lines 572-582:

Our approach validates the principle that synergy is achieved through the coordinated inhibition of compensatory or bypass pathways in the cell's signaling network. Pioneering network-based approaches, such as those by Cheng et al. (2019) (Cheng et al. 2019), have successfully utilized topological proximity to predict synergy, providing valuable insights into drug interactions based on the distance between drug targets and disease modules. Building on this foundation, our study employs Random Walk with Restart (RWR) to complement these proximity metrics by capturing the network-wide propagation of drug effects, revealing broader systemic impacts that local distance measures may overlook. Furthermore, a distinct innovation of our work is the adaptation of Graph-regularized Non-negative Matrix Factorization (GNMF) to the analysis of drug combinations. This approach identifies 'metagene' profiles, allowing distinct drug pairs to be grouped by their shared mechanism of action.
